# Supplementary material for: Clinical Significance of Glycolytic Metabolic Activity in Hepatocellular Carcinoma
Source: Cancers (Basel). 2022 Dec 28;15(1):186. doi: 10.3390/cancers15010186 (PMC9818850; doi:10.3390/cancers15010186)
Supplement: Supplementary file 1 [file cancers-15-00186-s001.zip › cancers-2036328-supplementary.pdf]

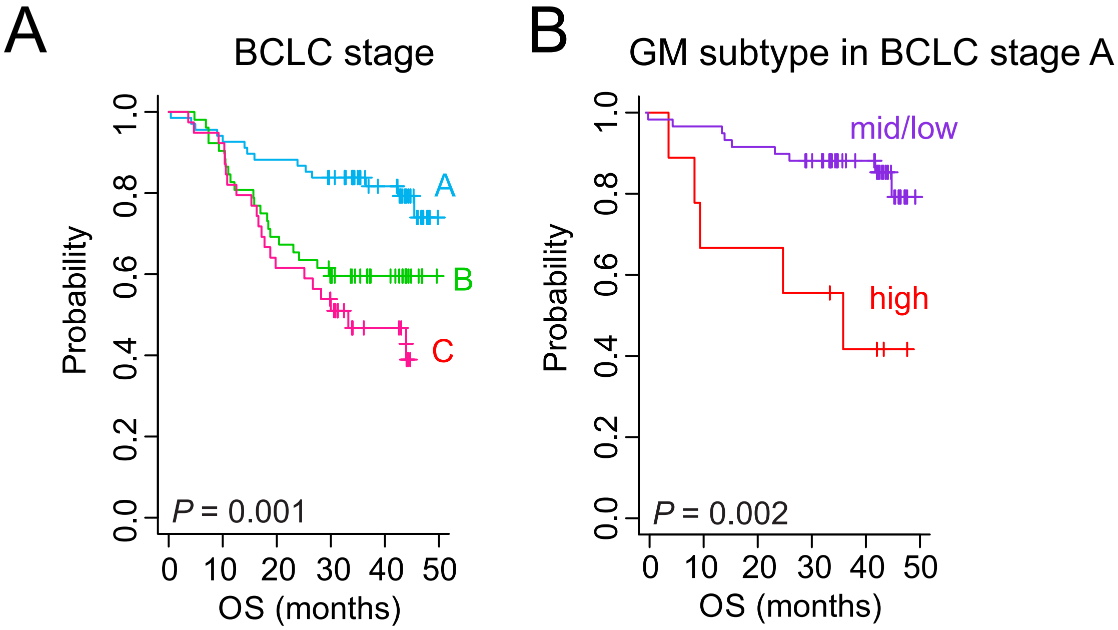

Supplementary Figure S1. Prognostic significance of glycolysis metabolic (GM) subtype in early-stage hepatocellular carcinoma (HCC).

(A) Kaplan-Meier plots of OS of HCC patients stratified by BCLC stage in Zhongshan cohort.
(B) Kaplan–Meier plots of OS of patients with BCLC stage A HCC stratified by GM subtype.

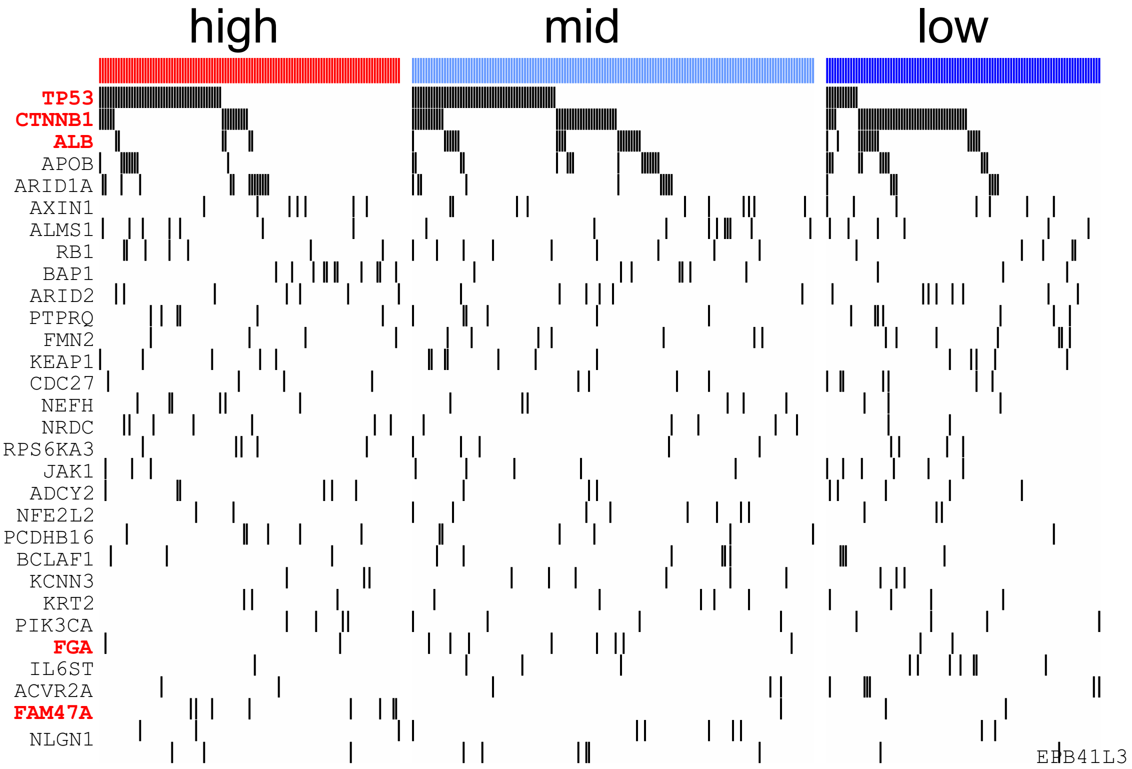

Supplementary Figure S2. Profile of somatic mutations in glycolysis metabolic subtypes in The Cancer Genome Atlas (TCGA) cohort.

Panel shows genes with statistically significant levels of mutation (MutSig suite, false discovery rate, 0.1 and >3% mutation rates) and mutation types are indicated in the legend at the top. Gene symbols in colored letters indicate genes significantly associated with the subtypes ($p < 0.05$).

"

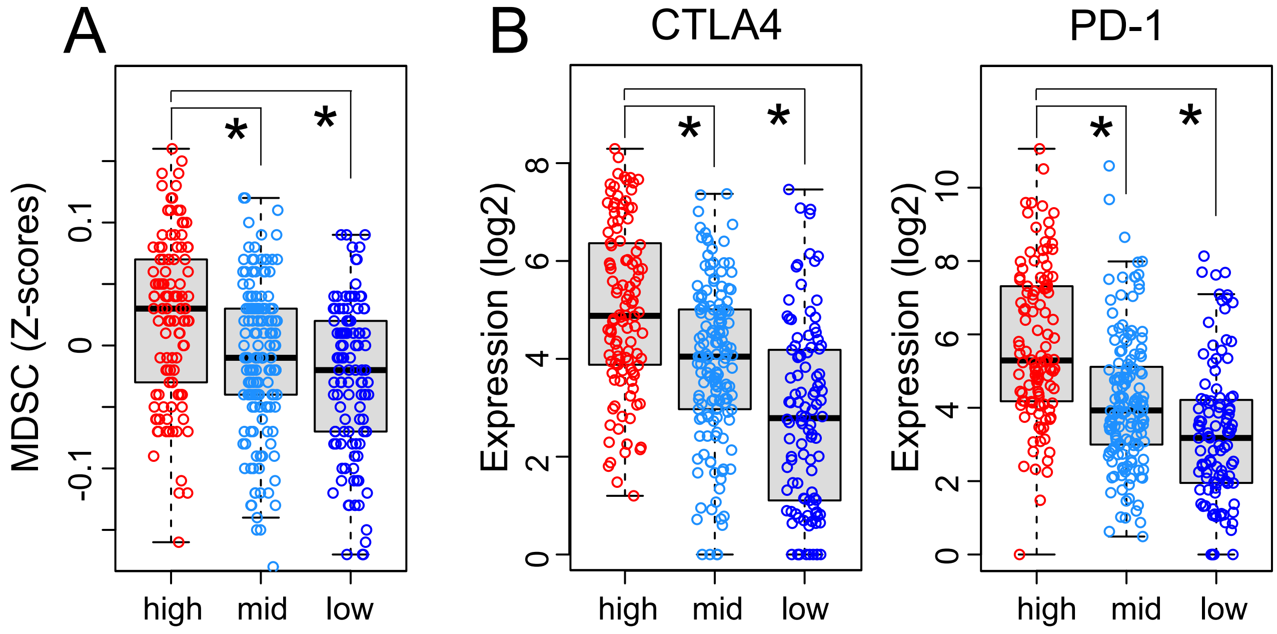

Supplementary Figure S3. Myeloid-derived suppressor cells (MDSCs) and expression of immune checkpoint genes in glycolysis metabolic (GM) subtypes.

(A) Box plots for estimated MDSC fractions in the three GM subtypes in The Cancer Genome Atlas Liver Hepatocellular Carcinoma (TCGA-LIHC) cohort (n = 371). Fraction of MDSC cells were estimated by tumor immune dysfunction and exclusion domain (TIDE) algorithm.

(B) Box plot for expression of immune checkpoints (CTLA-4 and PD-1) in three subtypes (TCGA-LIHC cohort, n = 371). In the box plots, the boundary of the box indicates the 25th to 75th percentile, and the black line within the box marks the mean. Whiskers above and below the box indicate the 10th and 90th percentiles. Each circle represents the Z-score of estimated cells or expression of indicated genes in each tumor. *p < 0.001 by Student t test.

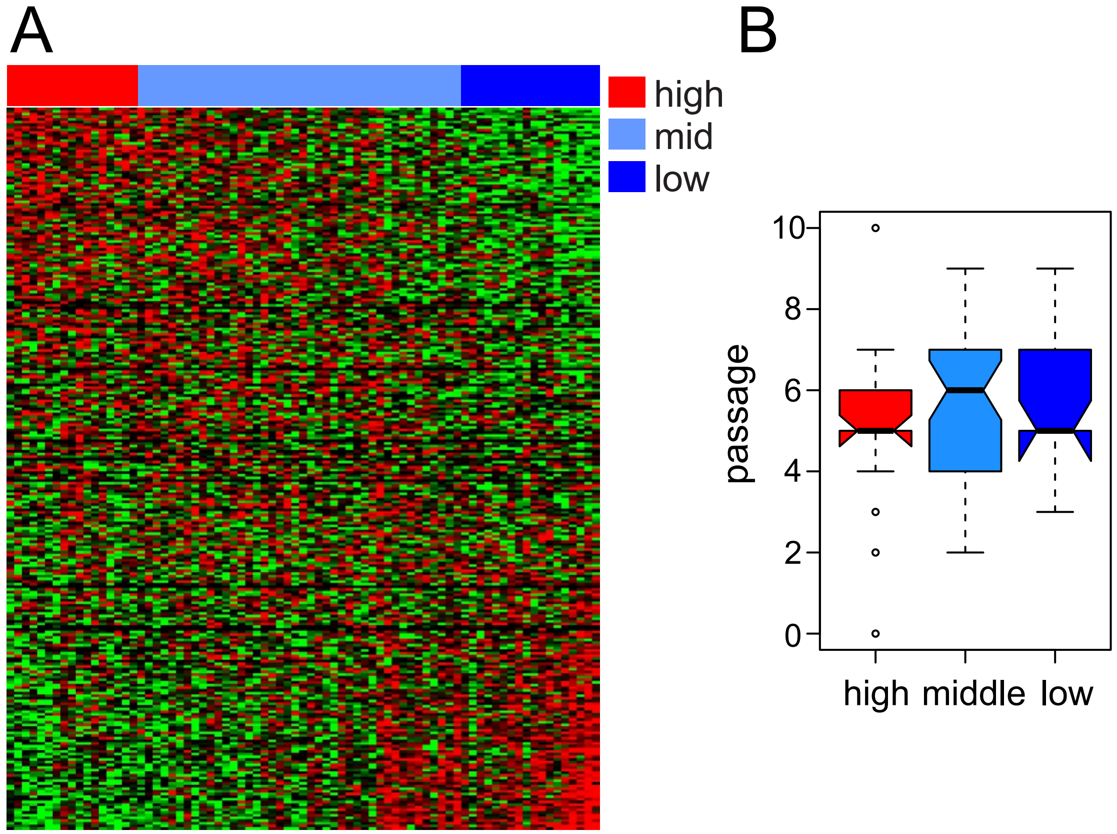

Supplementary Figure S4. Glycolysis metabolic (GM) subtypes in preclinical models for hepatocellular carcinoma (HCC).

(**A**) Expression of GM signatures in patient-derived xenograft hepatocellular carcinoma (PDX HCC) tumors (n = 77). GM subtypes were identified by Bayesian compound covariate prediction (BCCP) classifiers as described in the Methods. Data are presented in matrix format: each row represents an individual gene, and each column represents a sample. Each cell in the matrix represents the expression level of a gene feature in an individual tissue sample. Red and green indicate relatively high and low expression levels (z scores), respectively, as indicated in the log2-transformed scale bar.
(**B**) Number of passages of patient-derived xenograft (PDX) tumors for each subtype. In the box plots, the boundary of the box indicates the 25th to 75th percentile, and the black line within the box marks the mean. Whiskers above and below the box indicate the 10th and 90th percentiles on analysis. The area under the curve with 95% confidential interval was calculated using the ROC algorithm.

Supplementary Table S1. Summary of HCC gene expression data sets

Cohorts	Platform	Number of patients	Source
Fudan	Affymetrix U133A2	242	GEO, GSE14520
ZhongShan	RNA-seq	159	NODE, OEP000321
TCGA	RNA seq	371	https://xenabrowser.net/
Korea	Illumina V2, V4	188	GEO, GSE16757, GSE43619
Modena	Agilent-014850	78	GEO, GSE54236

TCGA, The Cancer Genome Project
GEO, Gene Expression Omnibus
NODE, National Omics Data Encyclopedia

Supplementary Table S2. Genes in glycolysis metabolic signature

Probe ID	*Symbol*	Ratio*
A_52_P235347	*Fgf21*	3.306889
A_55_P2052290	*Psat1*	2.79104
A_51_P408732	*Phgdh*	2.24597
A_55_P1973995	*Gm6756*	2.035042
A_55_P2168823		2.012508
A_55_P1958464		1.660607
A_55_P1986247	*Fut1*	1.546548
A_55_P2186929		1.53322
A_66_P124420		1.480867
A_55_P2016842	*Me1*	1.423426
A_51_P519251	*Nupr1*	1.368289
A_52_P94150	*Gm8096*	1.342803
A_51_P187625	*Cabp2*	1.207118
A_55_P2503096	*Plcxd1*	1.160097
A_51_P269216	*Atf5*	1.145222
A_55_P2009952	*Me1*	1.114235
A_55_P2837366	*Apoa4*	1.080756
A_55_P2744563	*Gpi1*	1.074947
A_66_P122536	*Gpi1*	1.073583
A_55_P2105858	*Atf5*	1.0505
A_55_P2022434	*Gpi1*	0.9983
A_55_P2030433	*Gpi1*	0.978678
A_66_P108003	*Espn*	0.966128
A_30_P01020769		0.947794
A_51_P322658	*Plcxd1*	0.927397
A_55_P1997651	*Espn*	0.924252
A_30_P01024405		0.915076
A_55_P2716236	*Rpia*	0.880812
A_52_P318040	*Acaca*	0.849078
A_55_P2019058	*Acaca*	0.824095
A_51_P176042	*Pklr*	0.809635
A_52_P184149	*Mthfd2*	0.795083
A_55_P2809133	*Aldh1l2*	0.777256
A_66_P140773	*Cd9*	0.769245
A_66_P114018		0.756437
A_52_P21659	*Pck2*	0.703669
A_51_P472249	*Slc7a7*	0.692694
A_52_P84096	*Pgd*	0.690209
A_55_P2732843	*Fads1*	0.679351
A_55_P2308283	*Gm16062*	0.663776

A_51_P440743	*Celsr1*	0.661031
A_52_P498193	*Aldh1l2*	0.660947
A_55_P2084703	*Acaca*	0.642496
A_51_P268529	*Csad*	0.629607
A_51_P394515	*Tkt*	0.628419
A_55_P2486634	*Fam124a*	0.620094
A_52_P468023	*Aldob*	0.608509
A_55_P2184009	*Rnd2*	0.594805
A_51_P215559	*BC021614*	0.594113
A_55_P2048498		0.581456
A_51_P279183	*Tkfc*	0.581304
A_52_P453508	*Dnase2a*	0.574504
A_55_P2105271	*Pdlim7*	0.562986
A_52_P608322	*Maff*	0.558096
A_51_P263302	*Rnf24*	0.555185
A_55_P2168643	*Khk*	0.553737
A_51_P404377	*Rnd2*	0.553122
A_51_P411757	*Ankrd13b*	0.529975
A_55_P2002578	*Ephx1*	0.528863
A_52_P72434	*Khk*	0.526146
A_51_P333111	*Aox1*	0.508208
A_52_P354248	*Lrrc28*	0.506538
A_51_P158073	*A230050P20Rik*	0.502894
A_51_P500996	*Fbxl20*	0.497964
A_51_P337708	*Ovgp1*	0.496422
A_66_P134775	*Pter*	0.485066
A_55_P2136561	*Gm10680*	0.48483
A_55_P1980287	*Birc3*	0.475394
A_52_P281941	*Zdhhc2*	0.473698
A_55_P2129261	*Arhgap36*	0.473341
A_55_P2113466		0.470494
A_55_P2050727	*Fcrl5*	0.465622
A_55_P2178800	*Ugt1a10*	0.463752
A_30_P01023097		0.45853
A_55_P2152836		0.452241
A_51_P307567	*Iqcb1*	0.451322
A_55_P2099358	*Cars*	0.449409
A_55_P2021049	*Ugp2*	0.449275
A_51_P314285	*Tmem86a*	0.4485
A_66_P137017	*Hcn3*	0.443441
A_55_P1973838	*Slc6a9*	0.436981
A_55_P2736170	*Ulk1*	0.435185
A_52_P151116	*Kcnab3*	0.434058

A_51_P150044	*Rps6ka1*	0.429668
A_30_P01026441		0.424583
A_52_P444804	*Cars*	0.420931
A_66_P118458	*Cars*	0.420157
A_55_P2740774	*Pter*	0.419565
A_52_P454994	*Pi4ka*	0.418878
A_65_P00756	*Hook2*	0.414291
A_55_P1969745	*Pitpnm1*	0.409716
A_55_P2087561	*Eno1b*	0.406007
A_30_P01019681		0.404895
A_52_P1197653		0.40454
A_52_P548202	*Gm10030*	0.403675
A_55_P2139256	*Rps6ka1*	0.401323
A_66_P119164	*Zfp354c*	0.399385
A_51_P125648	*Vwce*	0.390758
A_55_P2026218	*Igsf8*	0.390429
A_55_P1967648	*Flcn*	0.389751
A_55_P2739390	*Csad*	0.388446
A_52_P436628	*Vegfb*	0.388406
A_52_P309012	*Chmp4c*	0.382696
A_66_P131828		0.379113
A_55_P1952502	*Mapk7*	0.3779
A_51_P335419	*Csl*	0.377792
A_51_P276235	*Pnpla7*	0.377737
A_51_P251069	*Pter*	0.375225
A_52_P883557	*Slc30a10*	0.374418
A_66_P130241	*Slc25a1*	0.369429
A_55_P2827856	*Pard3b*	0.366044
A_51_P436878	*Sertad1*	0.361471
A_55_P2008327	*Eno1b*	0.361294
A_55_P2162269	*Bri3*	0.359169
A_55_P2723989		0.358941
A_55_P2740168	*Eno1b*	0.358288
A_66_P137219	*Elane*	0.356639
A_52_P225249	*Bri3*	0.35657
A_55_P2205156	*Aamdc*	0.355728
A_52_P349939	*Eno1*	0.355158
A_51_P507942	*Atp13a2*	0.351566
A_55_P2739629	*Lrrc28*	0.349497
A_55_P2713316	*Nmt2*	0.346834
A_52_P149743	*Rnf2*	0.346564
A_51_P448946	*Sertad2*	0.345783
A_52_P539310	*Serp2*	0.341252

A_55_P2038682		0.340964
A_55_P1983959	*Ftl1*	0.339479
A_52_P213483		0.338562
A_55_P1958097	*Eno1*	0.335055
A_51_P315682	*Igf2bp2*	0.330509
A_51_P321807	*4930519F09Rik*	0.330496
A_52_P161630	*St6gal1*	0.329352
A_66_P131389	*Ugt2b38*	0.328628
A_51_P479902	*Mars*	0.326502
A_30_P01026611		0.315772
A_52_P466741	*Hax1*	0.313973
A_66_P106702	*Cln6*	0.311912
A_55_P2078680	*Tpm1*	0.309905
A_51_P374337	*Flt3l*	0.306514
A_55_P2112225	*Pcyt2*	0.30563
A_55_P2042086	*Nrtn*	0.305257
A_30_P01021243		0.304692
A_55_P2091247		0.302881
A_55_P1957393		0.302845
A_55_P2737159	*App*	0.301928
A_51_P509098	*Asah1*	0.297552
A_55_P2740259		0.295255
A_51_P216595	*Usp28*	0.293527
A_66_P115035	*Il3ra*	0.28896
A_66_P105457	*Tpm1*	0.288897
A_51_P427934	*Megf8*	0.288375
A_30_P01021530		0.286648
A_66_P131205		0.285296
A_51_P462448	*Gpx4*	0.284898
A_51_P139848	*Wbp5*	0.283962
A_51_P374137	*Bco1*	0.282741
A_55_P2157992		0.280381
A_51_P446012	*Phf13*	0.280177
A_51_P394676	*Mef2d*	0.279434
A_55_P2093994	*Maged2*	0.278138
A_55_P2002819	*Rabgap1l*	0.277267
A_52_P434055	*Birc3*	0.276011
A_55_P2075657	*5330426L24Rik*	0.275436
A_51_P330428	*Eif4ebp1*	0.274429
A_55_P1961853	*Zfand3*	0.270468
A_55_P2923633	*Hax1*	0.269841
A_51_P294807	*Stom*	0.269216
A_55_P2831588	*Pcyt2*	0.266563

A_52_P16419	*Gpd1*	0.263928
A_55_P2043554	*Slc12a4*	0.262697
A_55_P2160204		0.26177
A_55_P2829619	*Yars*	0.260307
A_51_P450740	*Ptpn21*	0.258306
A_55_P1979684	*Rhoc*	0.257618
A_51_P352319	*Vps26b*	0.255445
A_66_P136570	*Actg1*	0.254491
A_30_P01030969		0.254139
A_52_P630632	*Rcbtb2*	0.253717
A_55_P2139385	*Gm12070*	0.253061
A_55_P2093649		0.251276
A_51_P507787	*Sergef*	0.251179
A_55_P2062733		0.249535
A_55_P2127265		0.247989
A_55_P1970299	*Mttp*	0.247271
A_55_P2024439	*Gaa*	0.247136
A_55_P2114118	*Cadm1*	0.243399
A_55_P2088014	*Cdc42ep4*	0.242768
A_55_P2494448	*Trip4*	0.242653
A_66_P137163	*Shisa6*	0.239713
A_52_P206526	*Gtf2ird2*	0.239484
A_51_P315555	*Nars*	0.237309
A_55_P2053456	*Gm6548*	0.237036
A_52_P244895	*Ncor1*	0.235602
A_66_P136076	*Cnot6l*	0.235433
A_66_P127314	*Tle2*	0.234539
A_55_P2220342	*Rabgap1l*	0.234334
A_51_P137947	*Ttc3*	0.234005
A_55_P2015337		0.233681
A_52_P315369	*Cyb5r1*	0.232172
A_52_P408858	*Brd1*	0.227727
A_51_P423668	*Fam160a2*	0.223704
A_52_P81980	*Micu1*	0.222538
A_55_P2504821	*Asah1*	0.222243
A_51_P493117	*Slc16a9*	0.222104
A_51_P452779	*Pygl*	0.221648
A_51_P347177	*Snx2*	0.221457
A_52_P453624	*Arl6ip6*	0.219283
A_51_P269078	*Habp4*	0.217077
A_52_P143477	*Tgoln1*	0.216998
A_55_P2255399	*F630048H11Rik*	0.216173
A_55_P2034893	*Socs7*	0.213881

A_55_P2798981	*Txnl4a*	0.213855
A_55_P1975021	*Rad18*	0.209665
A_55_P2739622	*Rcbtb2*	0.207661
A_55_P2845290	*Rsf1*	0.207201
A_51_P155458	*Dok7*	0.20502
A_52_P60194	*C4bp*	0.198989
A_66_P119815	*Erich1*	0.197268
A_30_P01023127		0.194538
A_52_P49601	*Fth1*	0.193202
A_52_P402663	*Ninl*	0.192005
A_51_P218805	*Gfer*	0.190407
A_66_P104044	*Rsf1*	0.189331
A_55_P2032222	*Tm9sf2*	0.186218
A_55_P2091245		0.185182
A_51_P259879	*Fkrp*	0.185045
A_52_P54238	*Lmf2*	0.180756
A_55_P2004469		0.179457
A_55_P2068330	*Cryzl1*	0.178351
A_55_P1958623	*Ncln*	0.173133
A_55_P1982872	*Naa10*	0.172352
A_66_P118719		0.169221
A_55_P1953136		0.168062
A_55_P2865960	*Habp4*	0.166986
A_55_P2070823	*Ino80d*	0.166574
A_51_P487360	*Hpcal1*	0.164636
A_55_P2059090	*Tagap*	0.162349
A_55_P2017774		0.153183
A_55_P2075013	*Tpt1*	0.152534
A_52_P363833	*Ybx1*	0.150486
A_55_P2128225	*Rpl18*	0.147725
A_55_P2904683	*Prdx1*	0.147029
A_55_P2163009	*Mapkapk5*	0.146402
A_55_P2136385	*Rps3a1*	0.142265
A_55_P1955287		0.141417
A_51_P446229	*Rpl18a*	0.141207
A_52_P436621	*Rpl18*	0.139623
A_55_P2015632	*Gm5766*	0.13762
A_51_P209444	*Rpl36al*	0.137039
A_55_P2909620	*Rpl7*	0.135561
A_66_P104479	*Rpl28*	0.134918
A_55_P1993813		0.13127
A_52_P667098	*Tpt1*	0.129922
A_55_P1960813		0.128495

A_55_P2178525	*Rpl26*	0.127901
A_52_P294675		0.126171
A_55_P2052634	*Rpl6*	0.124979
A_55_P2009267		0.123741
A_55_P1998848		0.123609
A_55_P2087589	*Rps11*	0.123604
A_51_P512002	*Rer1*	0.123063
A_55_P2018211		0.122657
A_55_P2170419		0.119063
A_55_P2051176		0.118148
A_55_P2155996		0.111762
A_55_P2012630		0.111109
A_55_P2125322		0.099588
A_52_P437321	*Timm17b*	0.09799
A_51_P184949	*F12*	0.091396
A_55_P2151899		0.079407
A_51_P234025	*Rbm5*	0.068312
A_55_P2817757	*Hip1r*	-0.02738
A_65_P07196		-0.07042
A_52_P640355	*Atp5h*	-0.09913
A_55_P2844945	*Atp5j2*	-0.10119
A_30_P01029682		-0.10349
A_55_P2152305	*Atp5h*	-0.10867
A_52_P289685	*Ssbp1*	-0.12505
A_51_P282706	*Hspd1*	-0.13885
A_55_P2035653	*Cep89*	-0.15237
A_30_P01022058		-0.15308
A_51_P338485	*Aldh6a1*	-0.15604
A_52_P165001	*Sec16a*	-0.15728
A_52_P108698	*LOC102631912*	-0.1622
A_51_P516615	*Ndufb10*	-0.16424
A_55_P2131068		-0.16539
A_55_P2482723	*Rnf181*	-0.16806
A_51_P429682	*Ebp*	-0.17057
A_52_P649064		-0.1709
A_55_P2130373	*Dcaf11*	-0.17243
A_55_P2006615	*Rbbp9*	-0.1743
A_55_P1994898	*Etfb*	-0.17708
A_65_P15689	*Mut*	-0.17771
A_55_P2742166	*Stambp*	-0.17808
A_55_P2106834	*Ndufs3*	-0.18248
A_55_P2055742	*Adtrp*	-0.19078
A_51_P198675	*Ttc36*	-0.19195

A_51_P504442	*Sf3a1*	-0.19195
A_55_P2739791	*Dlst*	-0.19348
A_55_P2792412	*Cox7b*	-0.19645
A_55_P2908509	*Sod2*	-0.19709
A_52_P499879	*Gstz1*	-0.19769
A_55_P2002117	*Kbtbd4*	-0.19976
A_51_P231687	*Ik*	-0.2003
A_51_P515452	*Fkbpl*	-0.20878
A_51_P249909	*Lect2*	-0.21388
A_55_P1972699	*Rars2*	-0.21444
A_55_P2091350	*ND4L*	-0.21544
A_52_P611213	*Dnajc28*	-0.21604
A_55_P2101231	*Aldh7a1*	-0.21693
A_51_P206225	*Uroc1*	-0.2177
A_30_P01023399		-0.21932
A_55_P2831148	*3110052M02Rik*	-0.22103
A_55_P2915367	*Gm4737*	-0.22121
A_52_P633163	*Mut*	-0.22225
A_66_P108095	*Zfp932*	-0.22644
A_55_P2502382	*Prmt10*	-0.22877
A_66_P116126	*Sdhb*	-0.23021
A_66_P138645	*Mir142hg*	-0.23034
A_55_P1988708	*Gstz1*	-0.23109
A_51_P362531	*Heatr1*	-0.23195
A_55_P2934413	*Acy1*	-0.23489
A_55_P2051859	*Golgb1*	-0.23507
A_51_P418526	*Sfxn1*	-0.2362
A_55_P2814732	*Slc25a53*	-0.23773
A_52_P216226	*Masp1*	-0.24589
A_55_P2817502	*Ppp1r10*	-0.25516
A_51_P114826	*Cdh13*	-0.25805
A_55_P2020896	*Parp16*	-0.26015
A_55_P2068496	*LOC102635467*	-0.26242
A_55_P2671706	*1700012D01Rik*	-0.26345
A_51_P443508	*Ppa1*	-0.26449
A_55_P2121827	*Dgcr2*	-0.26599
A_52_P407796	*Mdh1*	-0.26901
A_55_P2091631	*Coa4*	-0.26963
A_55_P2505730	*Erc1*	-0.27262
A_55_P2730491	*Cacfd1*	-0.28422
A_55_P2191630	*4931440J10Rik*	-0.28714
A_55_P2913723	*Swsap1*	-0.28753
A_52_P135572	*Dopey1*	-0.29192

A_51_P194853	*Sec14l4*	-0.29361
A_52_P434841	*Coa4*	-0.29881
A_55_P2796359	*Crem*	-0.29974
A_55_P2002033	*Wdr89*	-0.30153
A_55_P2736929	*Egf*	-0.30415
A_51_P464387	*Hspb8*	-0.30858
A_51_P511680	*Ttc38*	-0.30988
A_55_P2185821	*Cyp3a13*	-0.3114
A_55_P2056493	*Tk1*	-0.3144
A_52_P286360	*Otc*	-0.31467
A_51_P183051	*Upb1*	-0.31523
A_55_P2630393	*Slc38a4*	-0.31708
A_55_P1953899	*Mccc1*	-0.31758
A_51_P492676	*Sardh*	-0.32674
A_52_P640221	*Slc22a18*	-0.3307
A_51_P510437	*Slc25a15*	-0.33093
A_52_P569348	*Dbt*	-0.33111
A_55_P2822952	*Egf*	-0.33374
A_55_P2042698	*Hsd17b10*	-0.33824
A_55_P1953894	*Mccc2*	-0.3388
A_55_P2721817	*Aldh4a1*	-0.34119
A_52_P479269	*Sdc1*	-0.34565
A_55_P1960023	*Trmt5*	-0.35195
A_66_P125828		-0.35717
A_65_P20200	*Sdc1*	-0.35873
A_55_P2809848	*Adhfe1*	-0.3631
A_51_P335710	*Agpat6*	-0.36497
A_51_P413097	*Ints2*	-0.37112
A_55_P2051313	*Gstk1*	-0.38422
A_55_P2715658	*2010315B03Rik*	-0.39101
A_66_P105353	*Gcdh*	-0.40012
A_52_P1147453	*Fmo5*	-0.40784
A_51_P481238	*Dopey2*	-0.41115
A_55_P2067031	*Agxt2*	-0.41623
A_52_P394506	*Fam228a*	-0.41707
A_66_P102303	*Gpt*	-0.41967
A_52_P176160	*Ahcy*	-0.4216
A_51_P469153	*Fam214a*	-0.4218
A_51_P270635	*Gfpt1*	-0.4223
A_52_P450188	*Mtmr4*	-0.42542
A_52_P484838	*Rfxank*	-0.42819
A_66_P101632	*Afmid*	-0.43204
A_55_P2849189	*Slc7a2*	-0.43538

A_66_P118269	*Gpt*	-0.43653
A_55_P1983468	*Cflar*	-0.43696
A_55_P2044547	*2810416G20Rik*	-0.43976
A_52_P555629	*Zfp862-ps*	-0.4458
A_55_P2154242	*Gfpt1*	-0.45041
A_52_P436447	*Slc25a35*	-0.45185
A_55_P2071858	*Mgmt*	-0.46005
A_52_P20727	*Nhlrc1*	-0.46892
A_55_P2726885	*Fam46c*	-0.47104
A_55_P2485525	*Pxmp2*	-0.47139
A_51_P502119	*F11*	-0.47251
A_55_P2736854	*Meg3*	-0.47518
A_51_P425680	*Ivd*	-0.4816
A_65_P11840	*Ivd*	-0.48477
A_55_P2645225	*Pigr*	-0.49121
A_55_P1967325	*Fam19a5*	-0.5374
A_55_P2015495	*Abat*	-0.54153
A_51_P134142	*Cyp2c70*	-0.60538
A_55_P2907122	*Fam214a*	-0.66297
A_55_P2718683	*Dcxr*	-0.67771
A_52_P177352	*Lncbate1*	-0.68858
A_55_P1961014	*Selenbp1*	-0.69719
A_55_P2000022	*Ccdc151*	-0.69791
A_51_P268929	*Aox3*	-0.73938
A_55_P2077628	*Dcxr*	-0.7762
A_30_P01028919		-0.79858
A_51_P181312	*Dcxr*	-0.80138
A_55_P2115955	*Raet1e*	-0.83063
A_66_P125701	*Gm4673*	-0.8683
A_55_P2589727	*Aox3*	-0.87543
A_51_P467076	*Cyp2b9*	-1.28657
A_51_P469789	*Agxt*	-1.4456

*Ratio=fructose-glucose fed liver/tap water fed liver

Supplementary Table S3. Canonical pathways activated or deactivated in GM signature

Ingenuity Canonical Pathways	z-score	p-values
NRF2-mediated Oxidative Stress Response	2.646	2.8×10^{-7}
EIF2 Signaling	2.121	0.002
Glycolysis I	2	0.0003
Gluconeogenesis I	1.342	0.000017
tRNA Charging	1.342	0.00011
Cholecystokinin/Gastrin-mediated Signaling	1.342	0.016
mTOR Signaling	1.342	0.017
Ferroptosis Signaling Pathway	1.342	0.023
ERK5 Signaling	1	0.013
Xenobiotic Metabolism General Signaling Pathway	0.816	0.008
Sirtuin Signaling Pathway	0.632	0.0003
Nicotine Degradation III	0.447	0.00007
Serotonin Degradation	-0.447	0.001
Xenobiotic Metabolism AHR Signaling Pathway	-1.134	0.0001
Estrogen Receptor Signaling	-1.342	0.031
Xenobiotic Metabolism CAR Signaling Pathway	-1.508	0.000027
Xenobiotic Metabolism PXR Signaling Pathway	-1.508	0.000028
Oxidative Phosphorylation	-3	0.00001
